# Is surgery justified for 80-year-old or older intracranial meningioma patients? A systematic review

**DOI:** 10.1007/s10143-020-01282-7

**Published:** 2020-04-04

**Authors:** Ilari Rautalin, Mika Niemelä, Miikka Korja

**Affiliations:** Department of Neurosurgery, University of Helsinki and Helsinki University Hospital, P.O. Box 266, FI-00029 Helsinki, Finland

**Keywords:** Elderly, Intracranial meningioma, Surgery, Systematic review, 80 years old or older

## Abstract

Since the number of elderly people with intracranial meningiomas (IM) continues to rise, surgical treatment has increasingly become a considerable treatment option, even in very old (≥ 80 years old) meningioma patients. Since little is known about whether meningioma surgery in this age group is safe and justified, we conducted a systematic review to summarize the results of surgical outcomes in very old meningioma patients. We performed a systematic literature search in Pubmed, Cochrane Library, and Scopus databases. Primarily, we extracted 1-month and 1-year survival rates, and 1-year morbidity rates, as well as information about preoperative morbidity, operative complications, meningioma size, location, histology, and peritumoral edema. Quality of the included studies was evaluated by Cochrane Collaboration Handbook and Critical Appraisal Skills Program. From the 1039 reviewed articles, seven retrospective studies fulfilled our eligibility criteria. Motor deficits (27–65%) and mental changes (51–59%) were the most common indications for surgery. One-month and 1-year mortality rates varied between 0–23.5% and 9.4–27.3%, respectively. Most of the operated IM patients (41.2–86.5%) improved their performance during postoperative follow-up. Impaired preoperative performance and comorbidities were most commonly related to higher postoperative mortality. None of the studies fulfilled the criteria of high quality. Based on the evidence currently available, surgical treatment of very old IM patients seems to improve the performance of highly selected individuals. Given the rapid increase of the aging population, more detailed retrospective studies as well as prospective studies are needed to prove the outcome benefits of surgery in this patient group.

**Electronic supplementary material:**

The online version of this article (10.1007/s10143-020-01282-7) contains supplementary material, which is available to authorized users.

## Introduction

Intracranial meningioma (IM) is the most common primary intracranial tumor, accounting for over one-third of all intracranial tumors [25]. Since the growth rate of IMs is usually slow and the majority of tumors are benign ((World Health Organization (WHO) grades I–II)) [13], IMs may grow remarkably large before causing neurological or neuropsychological symptoms. As the life expectancy has rapidly increased in high-income countries [26], even octogenarians (80–90 years old) are often in relatively good condition, live independently at home, and have close to 10 years of life ahead of them [24]. Along with female sex, elderly age is among the most important risk factors for an increased chance of developing meningiomas [25]. As such, neurosurgical meningioma operations are likely to become more common even among very old patients (80 years old or older).

Increasing age is known to increase the risks of operative mortality and morbidity in cranial neurosurgery [18]. Indeed, three large register-based studies [1, 2, 23] have suggested that the operative risks for very old (i.e., ≥ 80 years) IM patients may increase as much as 15 times higher as compared with IM patients under 80 years old. On the other hand, two previous systematic reviews of elderly (≥ 65 years old) IM patients have shown that a proper patient selection process may decrease the operative risks, even in old IM patients [11, 17]. However, there is no overview for very old IM patients thus far.

We conducted a systematic review of studies including very old (≥ 80 years) IM patients who underwent a surgical procedure and were evaluated using one or more postoperative outcome measures. The main objective was to report short-term (1-month) and 1-year survival rates, as well as performance 1 year after surgery. Our primary hypothesis was that very old IM patients have similar 1-year outcomes as elderly meningioma patients; if so, this may be partly due to a highly specific patient selection process. Since it is likely that reports on very old meningioma patients are retrospective and include a limited number of patients, a descriptive systematic review (rather than meta-analysis) could provide a better overview of the topic.

## Materials and methods

The checklist of the Preferred Reporting Items for Systematic Review and Meta-Analyses (PRISMA) [21] guided our systematic review. A brief description of our research methods is presented below (see Supplementary Material 1 for detailed description).

### Study selection

We used the four-step PICO (patient, intervention, comparison, outcome) principle to frame a study question and eligibility criteria of our systematic literature search [9]. Three different databases, namely Pubmed, Scopus, and Cochrane Library, were used. To be included in the review, studies needed to consist of 80-year-old or older IM patients (patient) who underwent surgical tumor resection (intervention) and to assess postoperative morbidity or mortality (outcome). We excluded commentaries, case reports, case series (*n* < 5), letters, book chapters, reviews, and animal studies. Neither language nor publication year restrictions were used.

### Data extraction

In addition to general demographic characteristics, we extracted information about the following details: study design; indications for surgery; preoperative functional status and comorbidity; size, location, histology, and peritumoral edema of IMs; extent of tumor resection; short-term (1-month) morbidity and mortality; and 1-year morbidity, mortality, and recurrence rates. Furthermore, we tried to review the used prognostic factors and scales for complication-free outcome.

### Quality assessment

According to the Cochrane Collaboration Handbook [8] and Critical Appraisal Skills Program (CASP) [3], we used a domain-based evaluation by six individual domains to assess the quality of each included study: 1) IM characteristics (size, location, histology, and peritumoral edema), 2) preoperative functional status/morbidity, 3) extent of resection, 4) postoperative outcome (morbidity and mortality), and 5) prospective study design. Based on these domains, the studies included were classified as either low, unknown, or high risk of bias. To be classified as a high-quality study, each domain needed to be fulfilled and reported. If one or more domains were missing, we considered that study as high risk of bias, and subsequently classified it as low quality.

## Results

**Fig. 1:**
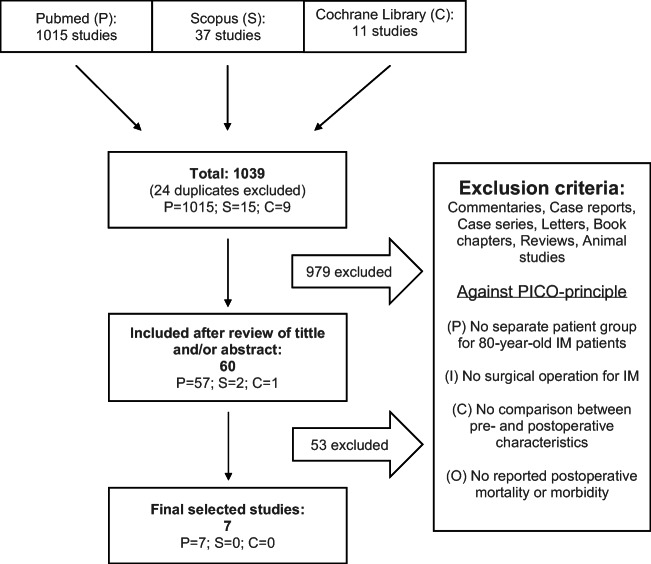
Study selection protocol

The study selection protocol is depicted in Fig. 1. From a total of 1039 articles, seven fulfilled the inclusion criteria [5, 6, 14, 15, 19, 20, 23]. All seven studies were retrospective, conducted between 1995 and 2018. Three of the studies were performed in Italy [5, 6, 15], two in France [19, 20], one in Norway [14], and one in the USA [23]. The latter [23] was based on a large national register (Table 1). Altogether, the studies included 308 operated IM patients, varying from 11 to 93 per study. There was an overrepresentation of females in each study (Table 1). One publication [19] was written in French.

**Table 1:** General characteristics of selected studies

First author, year, and reference	Country	Year	Case number	Age, median/mean (range)	% of male	Design
Mastronardi 1995 [15]	Italy	1995	17	82 (80–86)	23.5	Retrospective
D’Andrea 2005 [5]	Italy	2005	37	82 (80–86)	21.6	Retrospective
Riffaud 2007 [19]	France	2007	11	82 (81–87)	45.5	Retrospective
Sacko 2007 [20]	France	2007	74	82* (80–90)	36.5	Retrospective
Konglund 2013 [14]	Norway	2013	51	83.4* (80–90)	47.1	Retrospective
Dobran 2018 [6]	Italy	2018	25	81.9* (80–87)	32	Retrospective
Steinberg 2018 [23]	USA	2018	93	NR (> 80)	37.6	Retrospective, Register-based

*NR*, not reported; *, mean age

### Preoperative characteristics

#### Preoperative status and morbidity

All seven studies [5, 6, 14, 15, 19, 20, 23] used the American Society of Anesthesiology (ASA) scale [7] to assess preoperative physical status, which varied considerably among studies. Four studies [5, 6, 15, 19] included mostly healthy patients or patients with mild comorbidities (ASA classes I–II), whereas in three studies [14, 20, 23], most patients had severe systemic comorbidities (ASA classes III–V). In addition, six studies [5, 6, 14, 15, 19, 20] used the Karnofsky Performance Scale (KPS) [4] to describe IM patients’ preoperative functional status. In these studies, there was an equal representation of dependent (KPS 0–60) and independent (KPS 70–100) IM patients (Table 2). Six studies [5, 6, 14, 19, 20] reported indications for surgery resection. Motor deficits (27–65% in three studies), seizures (16–43% in four studies), and changed mental status (51–59% in three studies) were the most frequent symptoms leading to resection. Only one study [5] reported surgical treatment outcomes of asymptomatic IM patients (*n* = 4) (Table 2).

**Table 2:** Preoperative status and surgery indications

First author, year, and reference	Preoperative status				
	ASA class, *n* (%)		KPS score, *n* (%)		Indications of surgery/preoperative symptoms, *n* (%)
Mastronardi 1995 [15]	I	2 (11.8)	≥ 70	10 (59)	Impaired mental status: 10 (59)
	II	11 (64.7)	= 60	10 (59)	Asymptomatic: NR
	III	4 (23.5)	≤ 50	10 (59)	
	IV	0 (0.0)			
D’Andrea 2005 [5]	I	11 (33)	≥ 70	23 (62)	Headache: 27 (73)
	II	19 (50)	= 60	10 (27)	Impaired mental status: 19 (51)
	III	7 (17)	≤ 50	4 (11)	Impaired gait: 17 (46)
	IV	0 (0.0)			Seizures: 16 (43)
					Paresis: 11 (30)
					Sensory loss: 10 (27)
					Aphasia/dysphasia: 6 (16)
					Visual lost: 5 (13)
					Vertigo: 1 (3)
					Asymptomatic: 4 (11)
Riffaud 2007 [19]	I	0 (0.0)	≥ 80	≥ 80	Aphasia/dysphasia: 6 (55)
	II	8 (72.7)	≥ 80	≥ 80	Impaired mental status: 6 (55)
	III	8 (72.7)			Hemiparesis: 3 (27)
	IV	8 (72.7)			Intracranial hypertension: 1 (9)
					Visual loss: 1 (9)
					Impaired gait: 1 (9)
					Asymptomatic: 0 (0)
Sacko 2007 [20]	I	0 (0)	≥ 60	42 (56.8)	Motor deficits: 48 (65)
	II	22 (29.7)	≤ 50	32 (43.2)	Seizures: 32 (43)
	III	44 (59.4)			Intracranial hypertension: 16 (22)
	IV	8 (10.8)			Aphasia/dysphasia: 12 (16)
					Cerebellar symptoms: 12 (16)
					Asymptomatic: 0 (0)
Konglund 2013 [14]	I–II	17 (33.3)	≥ 80	21 (41)	Neurological deficits: 29 (54)
	III	30 (58.8)	60–70	21 (41)	Seizures: 22 (43)
	IV	4 (7.8)	≤ 50	9 (18)	Asymptomatic: NR
Dobran 2018 [6]	I	0	≥ 70	21 (84)	Neurological deficits: 21 (84)
	II	15 (60.0)	≥ 70	4 (16)	Seizures: 4 (16)
	III	10 (40.0)			Asymptomatic: 0 (0)
	IV	0			
Steinberger 2018 [23]	I–II	14 (15.0)	NR		NR
	III–V	79 (85.0)			

*ASA*, American Society of Anesthesiology scale; *KPS*, Karnofsky Performance Scale; *NR*, not reported

#### Characteristics of IMs

**Fig. 2:**
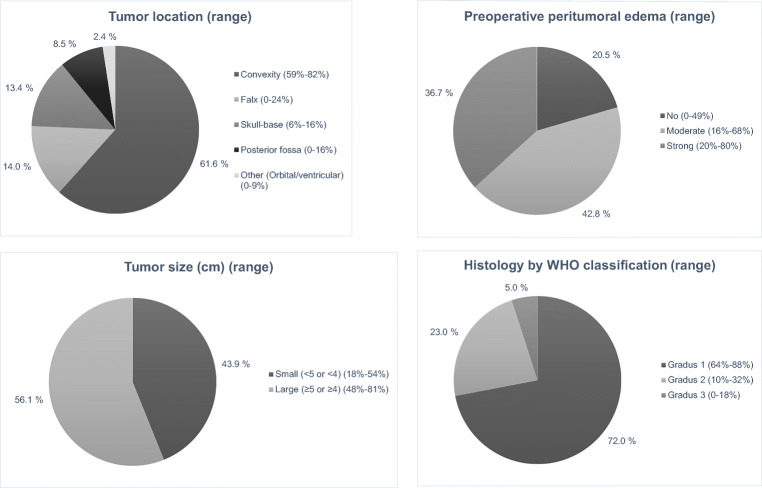
**a**–**d** Combined operated IM characteristics of all seven studies

Three studies [6, 19, 20] reported complete characteristics (size, location, histology, and peritumoral edema) of IMs. The majority of IMs were WHO grade I (range 64–88%), located in convexity (range 59–82%), had moderate or strong peritumoral edema (range 51–100%), and were categorized as large (at least 4 cm; range 46–81%) (Fig. 2–d).

### Postoperative characteristics

#### Tumor resection

**Fig. 3:**
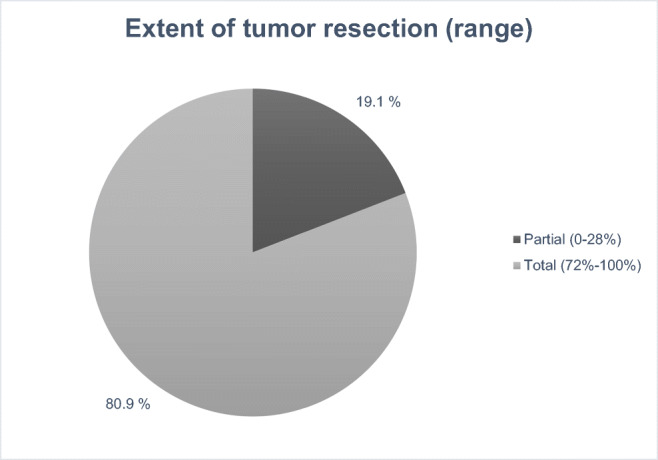
Combined surgery results (%) of all seven studies

All except one study [23] reported surgical results (Fig. 3). Total IM resection (Simpson grades I–II) was accomplished with the rate of 72–100%, depending on the study. No case with Simpson grade V resection (simple decompression with/without biopsy) was reported.

#### Postoperative mortality

**Fig. 4:**
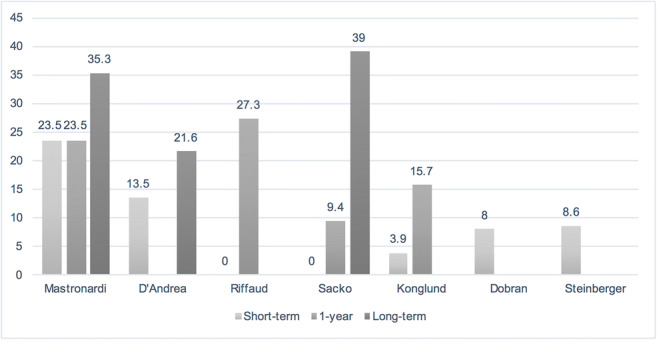
Short-term (within 30 days), 1-year and long-term (over 1 year) mortality rates (%)

All seven studies reported short-term (within 1 month after surgery) mortality rates (Fig. 4). The two earliest studies, partially from the same study population (Mastronardi et al. [15] in 1995, D’Andrea et al. [5] in 2005), reported the highest operative mortality rates of 23.5% and 13.5%, respectively. In more recent studies, mortality rates were lower (≤ 8.6%). Two studies [19, 20] did not report short-term mortality. One-year mortality rates were reported in four studies [14, 15, 19, 20], with figures ranging from 9.4 to 27.3%. In addition, three studies [5, 15, 20] reported long-term (over 1-year) follow-up results; for 60- [5], 96- [15], and 147-month [20] follow-ups, the mortality rates were 21.6%, 35.3%, and 39%, respectively.

#### Postoperative morbidity and recurrence

Overall postoperative complication rates varied between 9.1 and 31.2%, whereas the rate of major complications (death within the first month or a complication requiring re-operation) varied between 0 and 29.4%. The most common major complications were intracerebral hemorrhage (ICH), cardio-respiratory (CR) failure, and postoperative diffuse edema (PDE) (Table 3). According to five studies [5, 6, 15, 19, 20], the majority (41.2–86.5%) of operated IM patients improved their performance (KPS assessments) after the surgery. In addition, when analyzing only the first-year survivors, the proportions of patients with worsened KPS was minimal (0–15.4%). Four studies [5, 6, 15, 20] reported late recurrence rates, which varied from 0 to 12%. Recurrent meningiomas (*n* = 4) were operated in only one study [20] (Table 3).

**Table 3:** Postoperative morbidity and recurrence

First author, year, and reference	Postoperative complications, *n* (%)			Postoperative morbidity		Recurrence	
	Any	Major*	Other	Follow-up in months	Performance, KPS chance, *n* (%)	*N* (%)	Follow-up
Mastronardi 1995 [15]	5 (29.4)	CR failure: 1 (5.9) CSF leakage: 1 (5.9) PE: 1 (5.9) PDE: 2 (11.8) Any major: 5 (29.4)		12	Worse: 6 (35.3) Same: 4 (23.5) Better: 7 (41.2)	1 (5.9)	4 years
D’Andrea 2005 [5]	6 (16.2)	CR failure: 1 (2.7) CSF leakage: 1 (2.7) PE: 1 (2.7) PDE: 3 (8.1) Any major: 6 (16.2)		12	Worse: 5 (13.5) Same: 0 (0) Better: 32 (86.5)	1 (2.7)	4 years
Riffaud 2007 [19]	1 (9.1)	Any major: 0 (0)	Hemiplegia: 1 (9.1)	12	Worse: 1 (9.1) Same: 5 (45.4) Better: 5 (45.4)	0 (0)	NR
Sacko 2007 [20]	7 (9.4)	ICH: 1 (1.4) SDH: 1 (1.4) CSF leakage: 1 (1.4) Osteitis: 1 (1.4) Any major: 4 (5.4)	PE: 2 (2.7) ICH: 1 (1.4)	12	Worse: NR Same: NR Better: 39 (53)	4 (5.4)	15–147 months
Konglund 2013 [14]	NR	Deep surgical infection: 2 (3.9) Pneumonia: 1 (2.0) ICH: 3 (5.8) SDH: 1 (2.0) HCP: 1 (2.0) CR failure: 1 (2.0) PDE: 1 (2.0) Any major: 8 (15.7)	Neurological deficit: 8 (15.7) ICH: 4 (7.8) UTI: 9 (17.6) Pneumonia: 4 (7.8) CSF leakage: 1 (2.0)	NR	NR	NR	-
Dobran 2018 [6]	5 (20.0)	CR failure: 2 (8) Any major: 2 (8)	Wound infection: 1 (4) CSF leakage: 2 (8)	6	Mean KPS: Preoperatively: 74.3 Postoperatively: 82	3 (12)	3 years
Steinberger 2018 [23]	29 (31.2)	NR	Blood transfusion: 17 (18.3) Wound complication: 1 (1.1) Pulmonary complication: 7 (7.5) DVT/PE: 7 (7.5) UTI: 6 (6.5) CNS complications: 2 (2.2) Sepsis: 1 (1.1)	NR	NR	NR	NR

*CNS*, central nervous system; *CR*, cardio-respiratory; *CSF*, cerebrospinal fluid; *DVT*, deep vein thrombosis; *HCP*, hydrocephalus; *ICH*, intracerebral hemorrhage; *KPS*, Karnofsky Performance Scale; *NR*, not reported; *PDE*, postoperative diffuse edema; *PE*, pulmonary embolism; *SDH*, subdural hemorrhage; *UTI*, urinary tract infection

*Major complication = death within the first month or a complication requiring re-operation

#### Prognostic factors

Five studies [5, 6, 14, 15, 20] reported factors that related to adverse outcome, but only two [14, 20] used adjusted models (Table 4). In multivariate models, several factors including KPS score ≤ 80, moderate or strong peritumoral edema, male sex, and critical location were related to higher postoperative mortality rates. No predictive factors for postoperative morbidity were found. In univariate models, an ASA class ≥ 3 was most commonly associated with postoperative mortality, reported in five studies [5, 6, 14, 15, 20]. For morbidity, four studies [5, 6, 15, 20] reported the relation between an increased tumor size and increased complication rates or postoperative morbidity.

**Table 4:** Factors related to worse postoperative outcome (*p* < 0.05)

First author, year, and reference	Prognostic factors			
	For mortality		For morbidity or complications	
	Univariate	Multivariate	Univariate	Multivariate
Mastronardi 1995 [15]	ASA III KPS ≤ 60	NR	ASA III Tumor diameter	NR
D’Andrea 2005 [5]	ASA III KPS ≤ 70	NR	Tumor diameter Severe peritumoral edema Total surgical excision	NR
Riffaud 2007 [19]	NR	NR	NR	NR
Sacko 2007 [20]	Male sex KPS ≤ 50 Critical location ASA III Severe edema Low SKALE score	Critical location	Severe edema Tumor diameter Total removal	None
Konglund 2013 [14]	SKALE <8 Male sex ASA ≥ III Edema	KPS score Male sex Edema	None	None
Dobran 2018 [6]	Increasing ASA value Surgical time (> 240 min)	NR	Tumor diameter (> 4 cm) Surgical time (> 240 min)	NR
Steinberger 2018 [23]	NR	NR	NR	NR

*ASA*, American Society of Anesthesiology scale; *KPS*, Karnofsky Performance Scale; *NR*, not reported; *SKALE score*, sex, KPS, ASA, location, edema score

### Quality assessment

The domain-based evaluations are presented in Table 5. None of the included studies fulfilled our criteria for high quality. Major shortcomings and potential sources for various biases include retrospective design (all seven studies), limited reporting of IM characteristics (four studies [5, 14, 15, 23]), and limited outcome assessments (four studies [6, 14, 20, 23]) (Table 5).

**Table 5:** Quality assessment of selected studies. All studies were assigned to the low-quality category. + represents low risk of bias, − high risk of bias, and ? unknown risk of bias

First author, year, and reference	IM characteristics (size, histology, location, edema)	Preoperative morbidity	Extent of resection	Comprehensive outcome assessment (morbidity and mortality)	Prospective design
Mastronardi 1995 [15]	−	+	+	+	−
D’Andrea 2005 [5]	−	+	+	+	−
Riffaud 2007 [19]	+	+	+	+	−
Sacko 2007 [20]	+	+	+	−	−
Konglund 2013 [14]	?	+	+	−	−
Dobran 2018 [6]	+	+	+	−	−
Steinberger 2018 [23]	−	+	−	−	−

*IM*, intracranial meningioma

## Discussion

Based on the reviewed literature, seven retrospective studies have assessed surgical outcomes in 80-year-old or older IM patient. In the reviewed studies, 1-month and 1-year mortality varied between 0–23.5% and 9.4–27.3%, respectively, whereas all five studies [5, 6, 15, 19, 20] that reported pre- and postoperative performance levels (ranked by KPS) found that the majority of surgically treated IM patients improved in performance within 6 to 12 months after operation. Based on the available evidence, surgical treatment of IM patients 80 years old or older appears to be a relatively safe procedure, for which the benefits outweigh the potential risks in many patients—especially if preoperative risk assessment and patient selection processes are carefully conducted. Therefore, an increased age should perhaps not be used as a contraindication for meningioma surgery. On the other hand, very old male IM patients with severe comorbidities (ASA ≥ 3), impaired preoperative performance (KPS≤ 80), moderate or strong peritumoral edema, and critical tumor location (at the cranial base, near the large vessels, or in eloquent areas) may have a higher risk of postoperative mortality and morbidity.

All seven studies were conducted retrospectively with relatively low case numbers, as hypothesized. According to our quality analysis, shortcomings in IM characterization as well as in comprehensive outcome assessments may lead to a high risk of bias. None of the studies assessed whether the mortality rates were excessive, for example, by comparing the IM patients’ postoperative overall survival to the life expectancy of a comparable population. Ideally, the comparison group would contain age- and sex-matched non-operated IM patients with similar comorbidities, but even a comparison to a general population might help to elucidate surgical-related survival risks. Four studies reported 1-year mortality rates: 9.4% and 27.3% in France, 29.4% in Italy, and 15.7% in Norway. By comparison, current 1-year mortality rates in an age-matched population in these countries are 5.96% [12], 7.92% [10], and 8.19% [22], respectively. This may suggest that surgical treatment is associated with higher mortality in this patient group. However, since the operated patients had often progressive symptoms, which had jeopardized their independency, this comparison to the general population may overestimate the risks of surgery. Nevertheless, the treatment of asymptomatic patients should be carefully considered. The only study [23] that compared the surgical outcome of IM patients under 80 years old with those over 80 years old reported that age over 80 years was an independent risk factor for postoperative complications (OR = 2.4; 95% confidence interval (CI) 1.3–4.4)) and short-term mortality (OR = 15.7; CI 3.0–81.0). Thus, always aiming at radical removal at the expense of higher complication rates may not provide the best possible outcome, especially in this age group, since the growth rate of meningiomas is ultimately slow. In terms of functional recovery, five studies used KPS as a postoperative outcome assessment, but future studies could also consider simpler outcome measures such as ability to live at home after the surgery. This could perhaps provide more relevant information about IM patients’ dependency, recovery, and performance status.

Previously, two systematic reviews have examined surgical outcomes in elderly (65 years or older) IM patients. In 2017, the review by Ikawa et al. [11] included three studies [5, 14, 20] with very old (80-year-old or older) IM patients; in 2014, the review by Poon et al. [17] included two such studies [5, 20]. Despite the fact that these two reviews also included younger populations, their reported short-term (0–10.8% and 0–12%) and 1-year mortality rates (0–16.7% and 6.3–15.6%) were very similar to our findings. The same was true for complication rates (2.7–49.4% and 2.7–29.8%) and prognostic factors (ASA class, KPS, and peritumoral edema).

Due to the increased life expectancy, improved diagnostic modalities, and increased treatment options, the incidence of IMs in older populations has increased during recent decades [16]. Presently, the incidence of IMs in the general population is about one in every 12,500 people [16]. However, as the incidence rises with increasing age, the rate of occurrence increases to roughly one in 2000 [16] among very old (80-year-old and older) individuals—nearly twice as high as in 70-year-olds, and over five times higher than in 50-year-olds [16]. The physical condition and independence of these very old IM patients continue to improve, and these patients may retain their activity up to 90–95 years old. Therefore, this patient group needs to be studied in greater detail, especially in terms of the safety and benefits of surgical treatment.

While our review provides important insights into this topic, it is not without limitations. First, even though we performed our systematic literature search using three different databases, we may have missed some relevant studies. Second, due to the descriptive nature of the reviewed studies and the high risk of biases, we used only qualitative analyses to describe the current scientific evidence. Third, due to retrospective design of all reviewed studies, we believe that future studies with prospective data collection may provide more reliable information about the postoperative complications and their clinical impact on this patient group. Nevertheless, this systematic review provides the first overview of the scientific evidence for surgical treatment of 80-year-old or older IM patients and also guides future studies to avoid the critical shortcomings presented in the review. In addition, we believe that our review may aid in critical surgical decision-making processes.

## Conclusion

After a careful patient selection process, surgical removal of IM appears to be a relatively safe procedure, even in 80-year-old and older meningioma patients. However, prospective studies should confirm these findings by comparing postoperative outcomes, ideally to a matched IM patient group that undergo conservative treatment.

## Electronic supplementary material

ESM 1 (PDF 103 kb)
