# Supplementary material for: Is surgery justified for 80-year-old or older intracranial meningioma patients? A systematic review
Source: Neurosurg Rev. 2020 Apr 4;44(2):1061–9. doi: 10.1007/s10143-020-01282-7 (PMC8035086; doi:10.1007/s10143-020-01282-7)
Supplement: Supplementary file 1 — (PDF 103 kb) [file 10143_2020_1282_MOESM1_ESM.pdf]

Is surgery justified for 80-year-old or older intracranial meningioma patients? A systematic review

Ilari Rautalin, BM[1]; Mika Niemelä, MD, PhD[1]; Miikka Korja, MD, PhD[1]

[1]Department of Neurosurgery, University of Helsinki and Helsinki University Hospital, P.O. Box 266, FI-00029 Helsinki, Finland

Correspondence to Ilari Rautalin, Department of Neurosurgery, University of Helsinki, P.O. Box 266, FI-00029 Helsinki, Finland; E-mail address: ilari.rautalin@helsinki.fi; Telephone: +358 947187604; Fax: +358 947187616; ORCID 0000-0002-6283-0398

Journal name: Neurosurgical Review

Supplementary Material 1: Supplemental methods

Literature search

Pubmed

Preliminary search 28th January 2019

Search terms as written:

meningioma AND surgery AND (80 years or older)

Search terms expanded:

("meningioma"[MeSH Terms] OR "meningioma"[All Fields]) AND ("surgery"[Subheading] OR "surgery"[All Fields] OR "surgical procedures, operative"[MeSH Terms] OR ("surgical"[All Fields] AND "procedures"[All Fields] AND "operative"[All Fields]) OR "operative surgical procedures"[All Fields] OR "surgery"[All Fields] OR "general surgery"[MeSH Terms] OR ("general"[All Fields] AND "surgery"[All Fields]) OR "general surgery"[All Fields]) AND ((80[All Fields] AND years[All Fields]) OR older[All Fields])

Publications: 611

Final search 20th February 2019. (Updated 22nd May 2019)

Search terms as written:

meningioma AND (surgery OR operation) AND (aged, 80 and over[MeSH Terms] OR "80 years")

Search terms expanded:

("meningioma"[MeSH Terms] OR "meningioma"[All Fields]) AND (("surgery"[Subheading] OR "surgery"[All Fields] OR "surgical procedures, operative"[MeSH Terms] OR ("surgical"[All Fields] AND "procedures"[All Fields] AND "operative"[All Fields]) OR "operative surgical procedures"[All Fields] OR "surgery"[All Fields] OR "general surgery"[MeSH Terms] OR ("general"[All Fields] AND "surgery"[All Fields]) OR "general surgery"[All Fields]) OR ("surgical procedures, operative"[MeSH Terms] OR ("surgical"[All Fields] AND "procedures"[All Fields] AND "operative"[All Fields]) OR "operative surgical procedures"[All Fields] OR "operation"[All Fields])) AND ("aged, 80 and over"[MeSH Terms] OR "80 years"[All Fields])

Publications: 982 (1015 in update)

Scopus

Final search 20th February 2019. (Updated 22nd May 2019)

Search terms as written:

(meningioma AND (surgery OR operation) AND (elderly OR aged) AND ("80 years" OR "ninth decade" OR octogenarian))

Search terms expanded:

TITLE-ABS-KEY (meningioma AND (surgery OR operation) AND (elderly OR aged) AND ("80 years" OR "ninth decade" OR octogenarian))

Publications: 36 (37 in update)

Cochrane Library

Final search 20th February 2019. (Updated 22nd May 2019)

Search terms as written:

meningioma AND elderly

Search terms expanded:

'meningioma AND elderly in Title Abstract Keyword'

Publications: 9 (11 in update)

Search strategy

To formulate the specific study question, we used the recommended four-step PICO (Patient, Intervention, Comparison and Outcome) principle. The final study question formed was: Does surgical treatment of 80-year-old or older intracranial meningioma (IM) patients provide more beneficial changes in the performance and life years than conservative treatment?

Based on the study question, we performed a systematic literature search in three different databases: Pubmed, Scopus and Cochrane Library. To identify proper search terms, we used a preliminary search (performed on 28 January 2019) in the Pubmed database. By screening the index terms and keywords in potential publications, and in the studies of their reference lists, we were able to modify our search terms to include more comprehensively relevant studies. Thus, the final searches included both index terms and keywords in all three databases. Final searches were performed on 20 February 2019, and updated on 22 May 2019. The search strategy was planned together with all three authors and performed in English by the first author.

The PICO principle was also used to define the eligibility criteria to determine which studies would be included for further analyses. Specifically, in order to be included, studies needed to have 80-year-old or older IM patients (Patient) who underwent surgical tumor resection (Intervention), and to assess postoperative morbidity or mortality (Outcome). We excluded commentaries, case reports, case series (n < 5), letters, book chapters, reviews and animal studies. There were no restrictions based on language, publication year or publication area.

From the included studies, we extracted the following data: publication year, country, IM case number, median/mean age, age range, proportion of males and females, type of design, indications for surgery, preoperative functional status, comorbidities, size of IM, location of IM, histology of IM, peritumoral edema of IMs, extent of tumor resection, short-term (one-month) mortality, complications, short-term morbidity, long-term (more than one year) morbidity, long-term mortality, duration of follow-up, postoperative performance, postoperative independence, recurrence rates, surgical treatment of recurrent IMs and prognostic factors for postoperative morbidity and mortality.

Quality of included studies

According to the Cochrane Collaborator Handbook, we used a domain-based analysis to evaluate the quality of each included study; the checklist of Critical Appraisal Skills Program (CASP) guided the systematic evaluation. In total, we formed six individual domains to assess the potential source of different biases and methodological shortcomings in fundamental areas. All included studies were classified as low, unknown, or high risk of bias by each of the six domains. If the study fulfilled the low risk criteria in all six domains, it was classified as high quality. Otherwise, the study was categorized as low quality.

1. Meningioma characterization

Meningiomas may vary significantly in several tumor characteristics, which in turn may affect the surgical outcome. Therefore, in order to be classified as low risk of bias, studies needed to present complete characterization of reported meningiomas, including location, size, histology and the existence of peritumoral edema.

2. Preoperative morbidity

Preoperative physical condition and comorbidities may naturally affect the postoperative outcome. To be classified as low risk of bias, studies needed to assess preoperative morbidity by at least one proper measurement (e.g. American Society of Anesthesiologists (ASA) Score).

3. Extent of resection

The extent of surgery may also affect the postoperative outcome. For example, traumatic differences between complete removal (Simpson grade I) and simple decompression with/without biopsy can be enormous. Therefore, to be classified as low risk of bias, studies needed to report the extent of surgery.

4. Comprehensive outcome assessment

In addition to survival, assessment of physical condition, performance, independence and other possible complications or morbidities provides fundamental information about the safety of surgical treatments. Therefore, to be classified as low risk of bias, studies needed to assess not only postoperative mortality but also postoperative morbidity by at least one variable at one time point.

5. Prospective design

To provide possible risk factors for poor postoperative outcomes, or to provide high-quality evidence from surgical outcomes, retrospective studies have significant risks for different biases. Therefore, to be classified as low risk of bias, studies needed to be performed prospectively.
